# The genome sequence of the Scarce Umber, *Agriopis aurantiaria *(Hübner, 1799)

**DOI:** 10.12688/wellcomeopenres.19922.1

**Published:** 2023-10-13

**Authors:** Douglas Boyes, Peter O. Mulhair, University of Oxford and Wytham Woods Genome Acquisition Lab, Darwin Tree of Life Barcoding collective, Wellcome Sanger Institute Tree of Life programme, Wellcome Sanger Institute Scientific Operations: DNA Pipelines collective, Tree of Life Core Informatics collective, Darwin Tree of Life Consortium

**Affiliations:** 1 UK Centre for Ecology & Hydrology, Wallingford, England, UK; 2 University of Oxford, Oxford, England, UK

**Keywords:** Agriopis aurantiaria, Scarce Umber, genome sequence, chromosomal, Lepidoptera

## Abstract

We present a genome assembly from an individual male *Agriopis aurantiaria* (the Scarce Umber; Arthropoda; Insecta; Lepidoptera; Geometridae). The genome sequence is 485.4 megabases in span. The whole assembly is scaffolded into 30 chromosomal pseudomolecules, including the Z sex chromosome. The mitochondrial genome has also been assembled and is 15.44 kilobases in length. Gene annotation of this assembly on Ensembl identified 16,963 protein coding genes.

## Species taxonomy

Eukaryota; Metazoa; Eumetazoa; Bilateria; Protostomia; Ecdysozoa; Panarthropoda; Arthropoda; Mandibulata; Pancrustacea; Hexapoda; Insecta; Dicondylia; Pterygota; Neoptera; Endopterygota; Amphiesmenoptera; Lepidoptera; Glossata; Neolepidoptera; Heteroneura; Ditrysia; Obtectomera; Geometroidea; Geometridae; Ennominae; *Agriopis*; *Agriopis aurantiaria* (Hübner, 1799) (NCBI:txid104476).

## Background

*Agriopis aurantiaria* (Scarce Umber) is a moth from the family Geometridae with widespread distribution across Europe. It has documented increasing northward expansion across Fennoscandia, likely due to increasing temperatures ( Ammunét *et al.*, 2012; Jepsen *et al.*, 2011). Despite its name, this species is present throughout the United Kingdom and Ireland, with broad distribution and local abundance throughout much of Britain ( Randle *et al.*, 2019). It is most abundant in broadleaved woodland with mature trees, but can also be found in scrub and gardens near well-wooded regions. The larval foodplants include a variety of deciduous trees and shrubs, including *Quercus robur*, *Betula*, *Rosa*, and *Prunus padus* ( Robinson *et al.*, 2023). This species overwinters as an egg on the foodplant, with larvae emerging between April and June, and is found on the wing from October to December ( Waring *et al.*, 2017).

Perhaps the most striking feature of this species is the strong sexual dimorphism. Similar to other closely related species in Geometridae, males have normally developed wings while females are flightless due to almost completely vestigial wings (micropterous). Females can be found by searching tree trunks in the morning, where they rest ( Waring *et al.*, 2017). Wing reduction and flightless species have evolved many times within Lepidoptera, predominantly affecting females and species which are univoltine and have flight periods in the colder winter months ( Sattler, 1991), as is the case for *Agriopis aurantiaria*. A complete genome sequence will provide a basis for understanding how this trait has convergently evolved across Lepidoptera.

The genome of the scarce umber, *Agriopis aurantiaria*, was sequenced as part of the Darwin Tree of Life Project, a collaborative effort to sequence all named eukaryotic species in the Atlantic Archipelago of Britain and Ireland. Here we present a chromosomally complete genome sequence for *Agriopis aurantiaria*, based on one specimen from Wytham Woods, Oxfordshire.

## Genome sequence report

**Figure 1.**
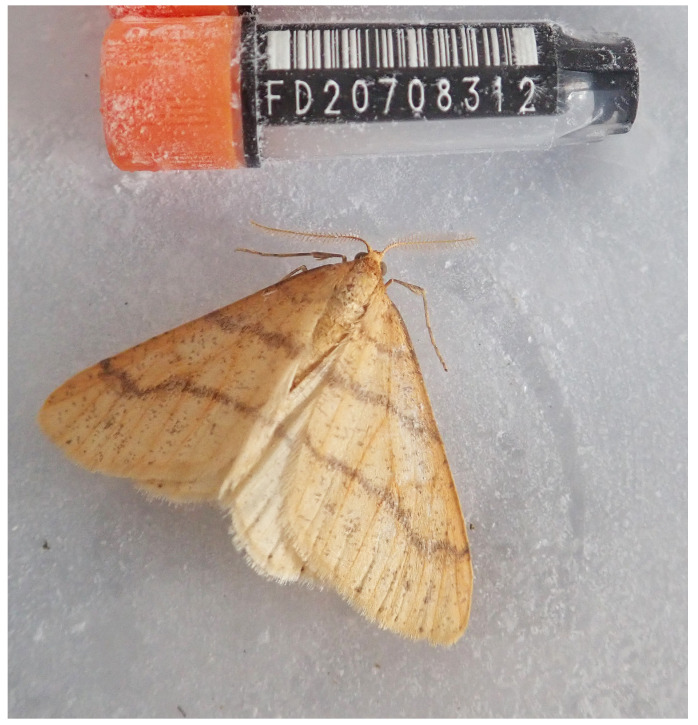
Photograph of the *Agriopis aurantiaria* (ilAgrAura1) specimen used for genome sequencing.

The genome was sequenced from one male *Agriopis aurantiaria* ( Figure 1) collected from Wytham woods, Oxfordshire (biological vice-county Berkshire), UK (51.77, –1.34). A total of 35-fold coverage in Pacific Biosciences single-molecule HiFi long reads and 90-fold coverage in 10X Genomics read clouds were generated. Primary assembly contigs were scaffolded with chromosome conformation Hi-C data. Manual assembly curation corrected 26 missing joins or mis-joins and removed 3 haplotypic duplications, reducing the assembly length by 0.44% and the scaffold number by 41.18%, and increasing the scaffold N50 by 1.59%.

**Figure 2.**
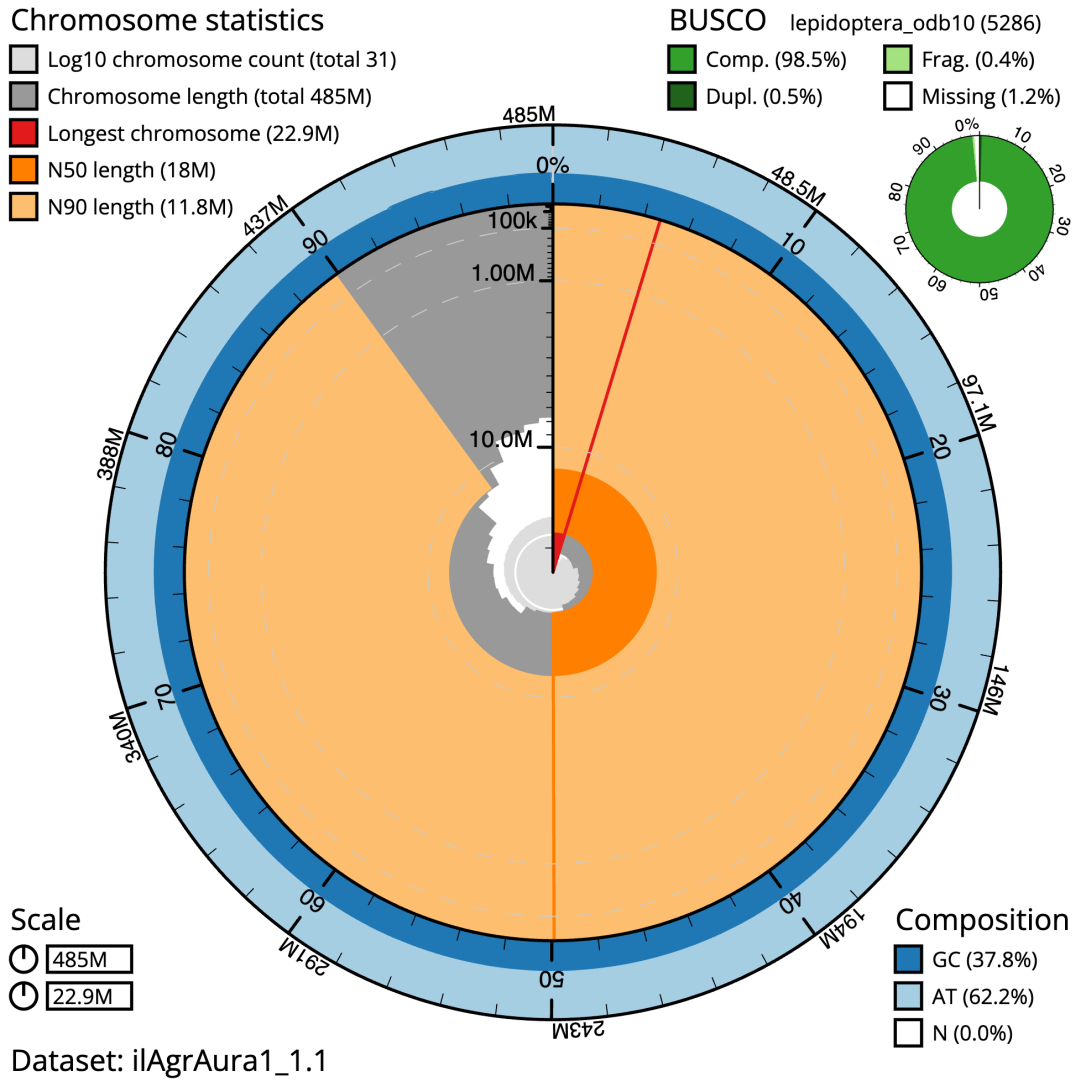
Genome assembly of *Agriopis aurantiaria*, ilAgrAura1.1: metrics. The BlobToolKit Snailplot shows N50 metrics and BUSCO gene completeness. The main plot is divided into 1,000 size-ordered bins around the circumference with each bin representing 0.1% of the 485,385,411 bp assembly. The distribution of scaffold lengths is shown in dark grey with the plot radius scaled to the longest scaffold present in the assembly (22,923,517 bp, shown in red). Orange and pale-orange arcs show the N50 and N90 scaffold lengths (18,049,239 and 11,825,588 bp), respectively. The pale grey spiral shows the cumulative scaffold count on a log scale with white scale lines showing successive orders of magnitude. The blue and pale-blue area around the outside of the plot shows the distribution of GC, AT and N percentages in the same bins as the inner plot. A summary of complete, fragmented, duplicated and missing BUSCO genes in the lepidoptera_odb10 set is shown in the top right. An interactive version of this figure is available at https://blobtoolkit.genomehubs.org/view/ilAgrAura1_1.1/dataset/ilAgrAura1_1.1/snail.

The final assembly has a total length of 485.4 Mb in 30 sequence scaffolds with a scaffold N50 of 18.0 Mb ( Table 1). The whole assembly sequence was assigned to 30 chromosomal-level scaffolds, representing 29 autosomes and the Z sex chromosome. Chromosome-scale scaffolds confirmed by the Hi-C data are named in order of size ( Figure 2– Figure 5; Table 2). While not fully phased, the assembly deposited is of one haplotype. Contigs corresponding to the second haplotype have also been deposited. The mitochondrial genome was also assembled and can be found as a contig within the multifasta file of the genome submission.

**Table 1.** Genome data for *Agriopis aurantiaria*, ilAgrAura1.1.

Project accession data		
Assembly identifier	ilAgrAura1.1	
Species	*Agriopis aurantiaria*	
Specimen	ilAgrAura1	
NCBI taxonomy ID	104476	
BioProject	PRJEB46315	
BioSample ID	SAMEA8603214	
Isolate information	ilAgrAura1, male: thorax (DNA sequencing), abdomen (RNA sequencing), head (Hi-C data)	
Assembly metrics *		*Benchmark*
Consensus quality (QV)	58.5	*≥ 50*
*k*-mer completeness	100%	*≥ 95%*
BUSCO **	C:98.5%[S:97.9%,D:0.5%],F:0.4%,M:1.2%,n:5,286	*C ≥ 95%*
Percentage of assembly mapped to chromosomes	100%	*≥ 95%*
Sex chromosomes	Z chromosome	*localised homologous pairs*
Organelles	Mitochondrial genome assembled	*complete single alleles*
Raw data accessions		
PacificBiosciences SEQUEL IIe	ERR6939239, ERR6808000	
10X Genomics Illumina	ERR6688508, ERR6688506, ERR6688509, ERR6688507	
Hi-C Illumina	ERR6688505	
PolyA RNA-Seq Illumina	ERR9435002	
Genome assembly		
Assembly accession	GCA_914767915.1	
*Accession of alternate haplotype*	GCA_914767795.1	
Span (Mb)	485.4	
Number of contigs	66	
Contig N50 length (Mb)	13.3	
Number of scaffolds	30	
Scaffold N50 length (Mb)	18.0	
Longest scaffold (Mb)	22.9	
Genome annotation		
Number of protein-coding genes	16,963	
Number of gene transcripts	17,104	

* Assembly metric benchmarks are adapted from column VGP-2020 of “Table 1: Proposed standards and metrics for defining genome assembly quality” from ( Rhie *et al.*, 2021). ** BUSCO scores based on the lepidoptera_odb10 BUSCO set using v5.3.2. C = complete [S = single copy, D = duplicated], F = fragmented, M = missing, n = number of orthologues in comparison. A full set of BUSCO scores is available at https://blobtoolkit.genomehubs.org/view/ilAgrAura1_1.1/dataset/ilAgrAura1_1.1/busco.

**Figure 3.**
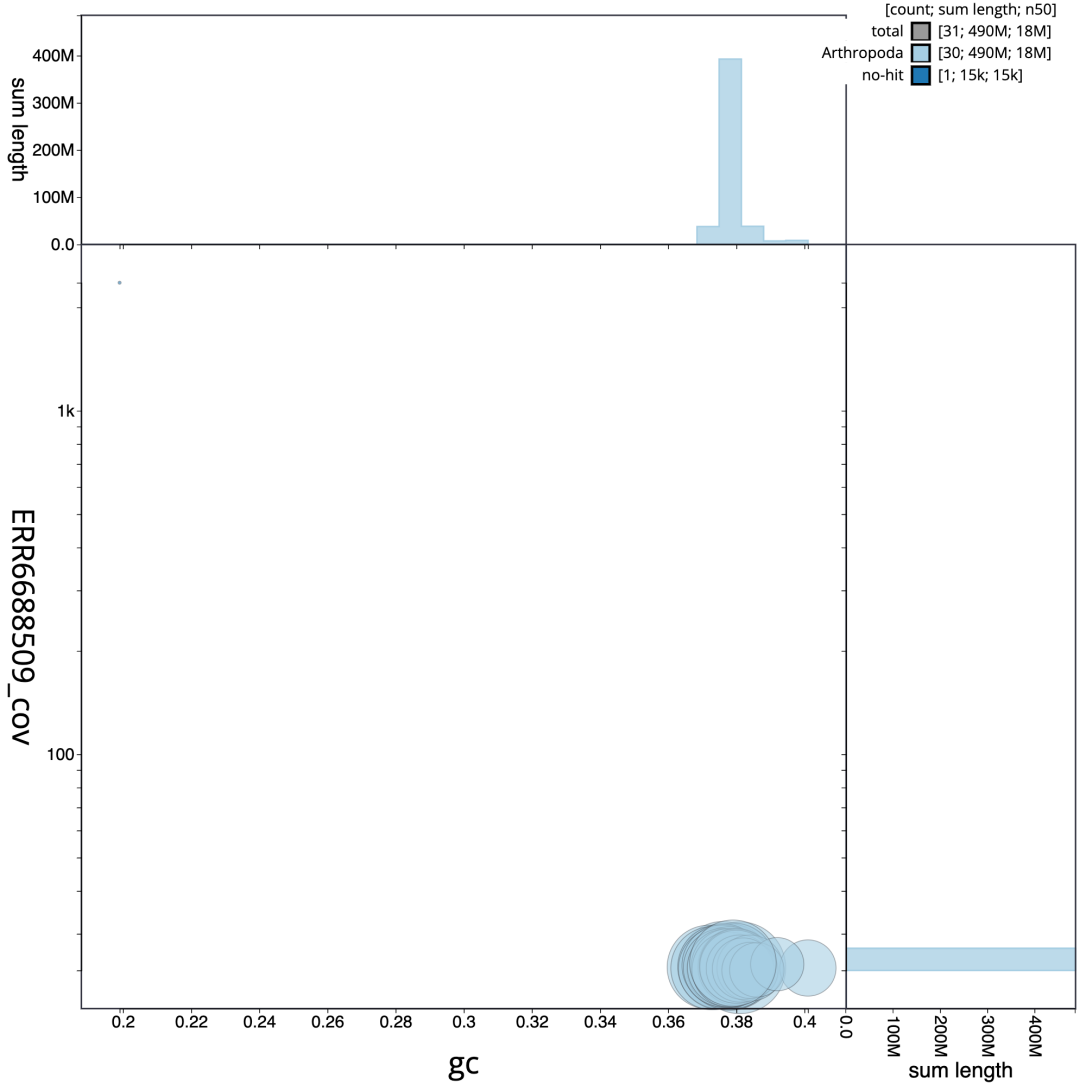
Genome assembly of *Agriopis aurantiaria*, ilAgrAura1.1: BlobToolKit GC-coverage plot. Scaffolds are coloured by phylum. Circles are sized in proportion to scaffold length. Histograms show the distribution of scaffold length sum along each axis. An interactive version of this figure is available at https://blobtoolkit.genomehubs.org/view/ilAgrAura1_1.1/dataset/ilAgrAura1_1.1/blob.

**Figure 4.**
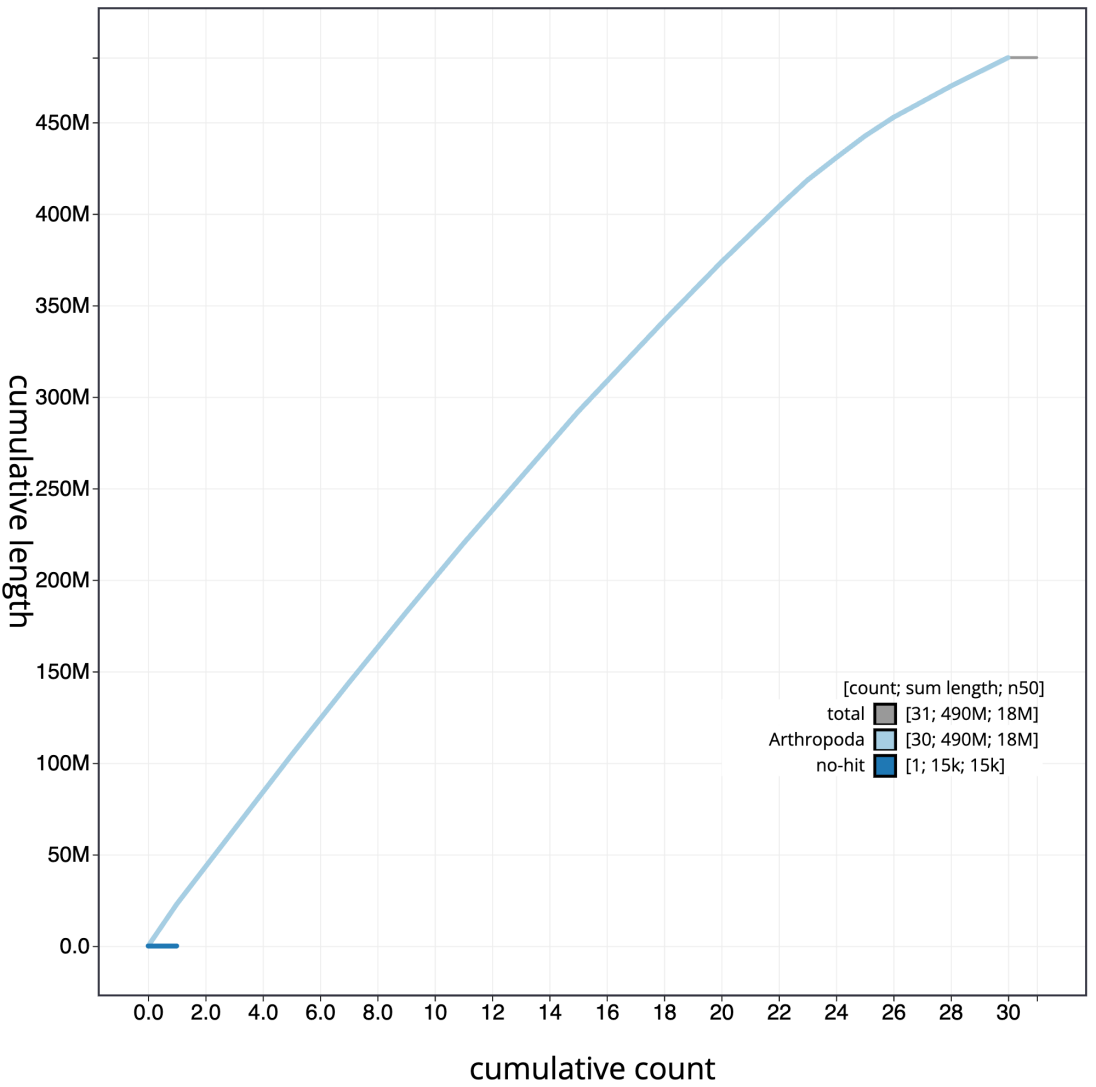
Genome assembly of *Agriopis aurantiaria*, ilAgrAura1.1: BlobToolKit cumulative sequence plot. The grey line shows cumulative length for all scaffolds. Coloured lines show cumulative lengths of scaffolds assigned to each phylum using the buscogenes taxrule. An interactive version of this figure is available at https://blobtoolkit.genomehubs.org/view/ilAgrAura1_1.1/dataset/ilAgrAura1_1.1/cumulative.

**Figure 5.**
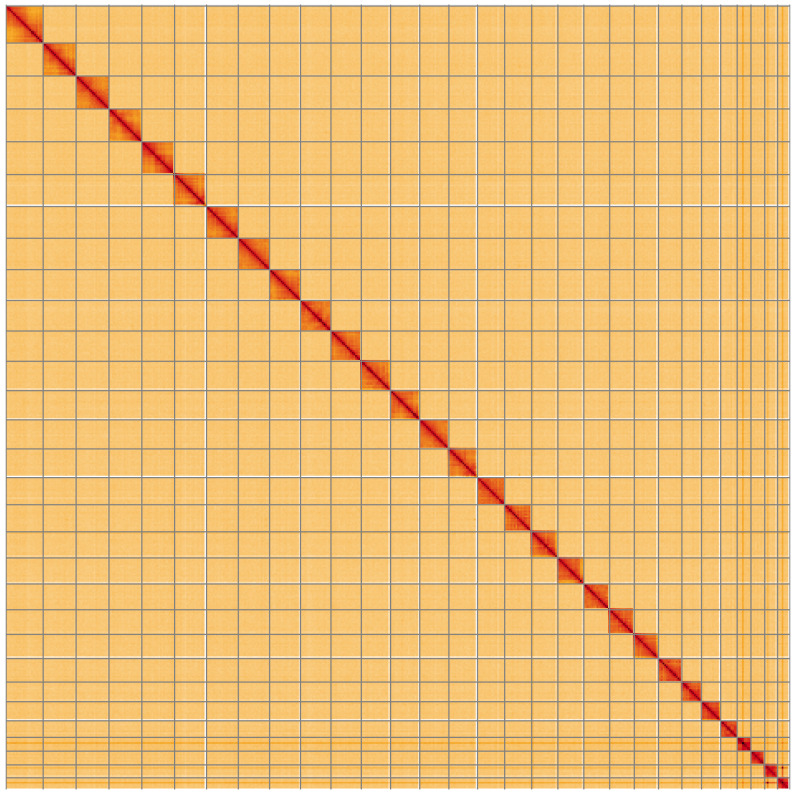
Genome assembly of *Agriopis aurantiaria*, ilAgrAura1.1: Hi-C contact map of the ilAgrAura1.1 assembly, visualised using HiGlass. Chromosomes are shown in order of size from left to right and top to bottom. An interactive version of this figure may be viewed at https://genome-note-higlass.tol.sanger.ac.uk/l/?d=Of-k_IrEQt6BBHNAbX4Cqw.

**Table 2.** Chromosomal pseudomolecules in the genome assembly of *Agriopis aurantiaria*, ilAgrAura1.

INSDC accession	Chromosome	Size (Mb)	GC%
OU611981.1	1	22.92	38.1
OU611982.1	2	20.4	37.9
OU611983.1	3	20.35	37.6
OU611984.1	4	20.32	37.8
OU611986.1	5	19.78	37.9
OU611987.1	6	19.66	37.6
OU611988.1	7	19.4	37.6
OU611989.1	8	19.13	37.2
OU611990.1	9	18.85	37.3
OU611991.1	10	18.78	37.5
OU611992.1	11	18.16	37.5
OU611993.1	12	18.05	37.7
OU611994.1	13	18.02	37.6
OU611995.1	14	17.77	37.8
OU611996.1	15	16.78	37.7
OU611997.1	16	16.74	37.6
OU611998.1	17	16.22	37.7
OU611999.1	18	16.06	37.8
OU612000.1	19	16.01	38
OU612001.1	20	15.26	37.8
OU612002.1	21	14.98	38
OU612003.1	22	14.48	38
OU612004.1	23	12.18	38.1
OU612005.1	24	11.83	38.4
OU612006.1	25	10.25	38.2
OU612007.1	26	8.53	40.1
OU612008.1	27	8.51	38.4
OU612009.1	28	7.99	38.6
OU612010.1	29	7.63	39.2
OU611985.1	Z	20.32	37.9
OU612011.1	MT	0.02	20.1

The estimated Quality Value (QV) of the final assembly is 58.5 with *k*-mer completeness of 100%, and the assembly has a BUSCO v5.3.2 completeness of 98.5% (single = 97.9%, duplicated = 0.5), using the lepidoptera_odb10 reference set ( *n* = 5,285).

Metadata for specimens, spectral estimates, sequencing runs, contaminants and pre-curation assembly statistics can be found at https://links.tol.sanger.ac.uk/species/104476.

## Genome annotation report

The *Agriopis aurantiaria* genome assembly (GCA_914767915.1) was annotated using the Ensembl rapid annotation pipeline ( Table 1; https://rapid.ensembl.org/Agriopis_aurantiaria_GCA_914767915.1/Info/Index). The resulting annotation includes 17,104 transcribed mRNAs from 16,963 protein-coding genes.

## Methods

### Sample acquisition and nucleic acid extraction

A male *Agriopis aurantiaria* (specimen ID Ox000991, individual ilAgrAura1) was collected from Wytham Woods, Oxfordshire (biological vice-county Berkshire), UK (latitude 51.77, longitude –1.34) on 2020-11-21, using a light trap. The specimen was collected and identified by Douglas Boyes (University of Oxford) and preserved on dry ice.

DNA was extracted at the Tree of Life laboratory, Wellcome Sanger Institute (WSI). The ilAgrAura1 sample was weighed and dissected on dry ice with head tissue set aside for Hi-C sequencing and abdomen tissue for RNA sequencing. Thorax tissue was disrupted using a Nippi Powermasher fitted with a BioMasher pestle. High molecular weight (HMW) DNA was extracted using the Qiagen MagAttract HMW DNA extraction kit. Low molecular weight DNA was removed from a 20 ng aliquot of extracted DNA using the 0.8X AMpure XP purification kit prior to 10X Chromium sequencing; a minimum of 50 ng DNA was submitted for 10X sequencing. HMW DNA was sheared into an average fragment size of 12–20 kb in a Megaruptor 3 system with speed setting 30. Sheared DNA was purified by solid-phase reversible immobilisation using AMPure PB beads with a 1.8X ratio of beads to sample to remove the shorter fragments and concentrate the DNA sample. The concentration of the sheared and purified DNA was assessed using a Nanodrop spectrophotometer and Qubit Fluorometer and Qubit dsDNA High Sensitivity Assay kit. Fragment size distribution was evaluated by running the sample on the FemtoPulse system.

RNA was extracted from abdomen tissue of ilAgrAura1 in the Tree of Life Laboratory at the WSI using TRIzol, according to the manufacturer’s instructions. RNA was then eluted in 50 μl RNAse-free water and its concentration assessed using a Nanodrop spectrophotometer and Qubit Fluorometer using the Qubit RNA Broad-Range (BR) Assay kit. Analysis of the integrity of the RNA was done using Agilent RNA 6000 Pico Kit and Eukaryotic Total RNA assay.

### Sequencing

Pacific Biosciences HiFi circular consensus and 10X Genomics read cloud DNA sequencing libraries were constructed according to the manufacturers’ instructions. Poly(A) RNA-Seq libraries were constructed using the NEB Ultra II RNA Library Prep kit. DNA and RNA sequencing was performed by the Scientific Operations core at the WSI on Pacific Biosciences SEQUEL II (HiFi), Illumina HiSeq 4000 (RNA-Seq) and Illumina NovaSeq 6000 (10X) instruments. Hi-C data were also generated from head tissue of ilAgrAura1 using the Arima2 kit and sequenced on the Illumina NovaSeq 6000 instrument.

### Genome assembly, curation and evaluation

Assembly was carried out with Hifiasm ( Cheng *et al.*, 2021) and haplotypic duplication was identified and removed with purge_dups ( Guan *et al.*, 2020). One round of polishing was performed by aligning 10X Genomics read data to the assembly with Long Ranger ALIGN, calling variants with FreeBayes ( Garrison & Marth, 2012). The assembly was then scaffolded with Hi-C data ( Rao *et al.*, 2014) using SALSA2 ( Ghurye *et al.*, 2019). The assembly was checked for contamination and corrected as described previously ( Howe *et al.*, 2021). Manual curation was performed using HiGlass ( Kerpedjiev *et al.*, 2018) and Pretext ( Harry, 2022). The mitochondrial genome was assembled using MitoHiFi ( Uliano-Silva *et al.*, 2023), which runs MitoFinder ( Allio *et al.*, 2020) or MITOS ( Bernt *et al.*, 2013) and uses these annotations to select the final mitochondrial contig and to ensure the general quality of the sequence.

A Hi-C map for the final assembly was produced using bwa-mem2 ( Vasimuddin *et al.*, 2019) in the Cooler file format ( Abdennur & Mirny, 2020). To assess the assembly metrics, the *k*-mer completeness and QV consensus quality values were calculated in Merqury ( Rhie *et al.*, 2020). This work was done using Nextflow ( Di Tommaso *et al.*, 2017) DSL2 pipelines “sanger-tol/readmapping” ( Surana *et al.*, 2023a) and “sanger-tol/genomenote” ( Surana *et al.*, 2023b). The genome was analysed within the BlobToolKit environment ( Challis *et al.*, 2020) and BUSCO scores ( Manni *et al.*, 2021; Simão *et al.*, 2015) were calculated.

Table 3 contains a list of relevant software tool versions and sources.

**Table 3.** Software tools: versions and sources.

Software tool	Version	Source
BlobToolKit	4.0.7	https://github.com/blobtoolkit/blobtoolkit
BUSCO	5.3.2	https://gitlab.com/ezlab/busco
FreeBayes	1.3.1-17-gaa2ace8	https://github.com/freebayes/freebayes
Hifiasm	0.15.3	https://github.com/chhylp123/hifiasm
HiGlass	1.11.6	https://github.com/higlass/higlass
Long Ranger ALIGN	2.2.2	https://support.10xgenomics.com/genome-exome/software/pipelines/latest/advanced/other-pipelines
Merqury	MerquryFK	https://github.com/thegenemyers/MERQURY.FK
MitoHiFi	2	https://github.com/marcelauliano/MitoHiFi
PretextView	0.2	https://github.com/wtsi-hpag/PretextView
purge_dups	1.2.3	https://github.com/dfguan/purge_dups
SALSA	2.2	https://github.com/salsa-rs/salsa
sanger-tol/genomenote	v1.0	https://github.com/sanger-tol/genomenote
sanger-tol/readmapping	1.1.0	https://github.com/sanger-tol/readmapping/tree/1.1.0

### Genome annotation

The BRAKER2 pipeline ( Brůna *et al.*, 2021) was used in the default protein mode to generate annotation for the *Agriopis aurantiaria* assembly (GCA_914767915.1) in Ensembl Rapid Release.

### Wellcome Sanger Institute – Legal and Governance

The materials that have contributed to this genome note have been supplied by a Darwin Tree of Life Partner. The submission of materials by a Darwin Tree of Life Partner is subject to the **‘Darwin Tree of Life Project Sampling Code of Practice’**, which can be found in full on the Darwin Tree of Life website here. By agreeing with and signing up to the Sampling Code of Practice, the Darwin Tree of Life Partner agrees they will meet the legal and ethical requirements and standards set out within this document in respect of all samples acquired for, and supplied to, the Darwin Tree of Life Project. 

Further, the Wellcome Sanger Institute employs a process whereby due diligence is carried out proportionate to the nature of the materials themselves, and the circumstances under which they have been/are to be collected and provided for use. The purpose of this is to address and mitigate any potential legal and/or ethical implications of receipt and use of the materials as part of the research project, and to ensure that in doing so we align with best practice wherever possible. The overarching areas of consideration are:

• Ethical review of provenance and sourcing of the material

• Legality of collection, transfer and use (national and international) 

Each transfer of samples is further undertaken according to a Research Collaboration Agreement or Material Transfer Agreement entered into by the Darwin Tree of Life Partner, Genome Research Limited (operating as the Wellcome Sanger Institute), and in some circumstances other Darwin Tree of Life collaborators.

## Data Availability

European Nucleotide Archive: *Agriopis aurantiaria* (scarce umber). Accession number PRJEB46315; https://identifiers.org/ena.embl/PRJEB46315. ( Wellcome Sanger Institute, 2021) The genome sequence is released openly for reuse. The *Agriopis aurantiaria* genome sequencing initiative is part of the Darwin Tree of Life (DToL) project. All raw sequence data and the assembly have been deposited in INSDC databases. Raw data and assembly accession identifiers are reported in Table 1.

## Author information

Members of the University of Oxford and Wytham Woods Genome Acquisition Lab are listed here: https://doi.org/10.5281/zenodo.4789928.

Members of the Darwin Tree of Life Barcoding collective are listed here: https://doi.org/10.5281/zenodo.4893703.

Members of the Wellcome Sanger Institute Tree of Life programme are listed here: https://doi.org/10.5281/zenodo.4783585.

Members of Wellcome Sanger Institute Scientific Operations: DNA Pipelines collective are listed here: https://doi.org/10.5281/zenodo.4790455.

Members of the Tree of Life Core Informatics collective are listed here: https://doi.org/10.5281/zenodo.5013541.

Members of the Darwin Tree of Life Consortium are listed here: https://doi.org/10.5281/zenodo.4783558.
