# Supplementary material for: The genome sequence of the Scarce Umber, Agriopis aurantiaria (Hübner, 1799)
Source: Wellcome Open Res. 2023 Oct 13;8:463. [Version 1] doi: 10.12688/wellcomeopenres.19922.1 (PMC11109570; doi:10.12688/wellcomeopenres.19922.1)
Supplement: Peer review history

# Reviewer report: Reviewer response for version 1

**DOI:** 10.21956/wellcomeopenres.22061.r81131

**Published:** 2024-05-14

**Authors:** Jan Veenstra

**Affiliations:** 1 University of Bordeaux, Pessac, France

This is yet another high quality DNA assembly from the Wellcome Sanger Institute. High quality says it all. There is really nothing here to criticize.

 Feeling obliged to make a few comments, I add the  following. It would have been nice to have a picture illustrating the sexual dimorphism of this species. It is very satisfying to see not only Pacific Biosciences HIFI sequences but also abundant short read illumina sequences as well at some RNAseq data. The latter in this case is exclusively from a single male and lack the brain, a tissue which as a neuroscientist I find particularly interesting. These comments should not give the impression that I am negative, that would really wrong. As stated in the first line of this review, this is high quality and as such a welcome addition to the data bank. It would be great if someone were to use this and other genome assemblies to identify genes that might be responsible for the sexual dimorphism.

Are sufficient details of methods and materials provided to allow replication by others?

Yes

Is the rationale for creating the dataset(s) clearly described?

Yes

Are the datasets clearly presented in a useable and accessible format?

Yes

Are the protocols appropriate and is the work technically sound?

Yes

Reviewer Expertise:

Insect neuropeptides

I confirm that I have read this submission and believe that I have an appropriate level of expertise to confirm that it is of an acceptable scientific standard.

# Reviewer report: Reviewer response for version 1

**DOI:** 10.21956/wellcomeopenres.22061.r68731

**Published:** 2023-10-25

**Authors:** Benjamin Buer

**Affiliations:** 1 Bayer AG, Crop Science Division, Germany

The Data Note describes the genome sequencing and annotation of the Scarce Umber ( *Agriopis aurantiaria*). The genome, derived from an individual male, comprises 29 chromosomes plus the sex and the mitochondrial genome. As Scarce Umber has strong sexual dimorphism, the reference genome may be relevant to further understand the evolution of this feature in lepidopteran species in future.

 The standard metrics of the genome are of high quality and the majority of methods are described well but some details of parameters may be helpful to reproduce the analysis. For the assembly, MitoHiFi was used, however the specific algorithm (MitoFinder or MITOS, or a consensus of both) was not clearly mentioned.

 The genome was produced by assembling reads of an individual male collected in the UK using PacBio and Illumina sequencing data from thorax tissue and Hi-C technology for scaffolding from head tissue. In addition, the genome was annotated using BRAKER2 pipeline, however the use of the RNA sequencing data that was generated from abdomen data remains unclear in this process and may be further elaborated.

Are sufficient details of methods and materials provided to allow replication by others?

Partly

Is the rationale for creating the dataset(s) clearly described?

Yes

Are the datasets clearly presented in a useable and accessible format?

Yes

Are the protocols appropriate and is the work technically sound?

Yes

Reviewer Expertise:

Bioinformatics

I confirm that I have read this submission and believe that I have an appropriate level of expertise to confirm that it is of an acceptable scientific standard.
